# From "dose erythema" to FLASH radiotherapy: impacts on clinical practice

**DOI:** 10.1590/1806-9282.2024S130

**Published:** 2024-06-07

**Authors:** Heloisa de Andrade Carvalho, Geovanne Pedro Mauro, Marcus Simões Castilho

**Affiliations:** 1 Universidade de São Paulo, Faculdade de Medicina, Hospital das Clínicas HCFMUSP, Department of Radiology and Oncology, Radiotherapy Division (INRAD and ICESP) – São Paulo (SP), Brazil.; 2 Radiocare-Hospital Felício Rocho and Oncobio – Belo Horizonte (MG), Brazil.

## BRIEF HISTORY

After the discovery of X-rays in 1985 and radioactivity in 1986, the potential of these types of radiation was immediately recognized as an essential tool in medicine. At first, the biological effects and dose magnitudes of radiation were unknown; thus, the biological marker of the therapeutic dose was the redness of the skin after some time of exposure. This kind of dose measurement was known as "dose-erythema" based on which the therapeutic effect and tolerance of the tissues were estimated. During these first years, radiation was used in a variety of scenarios of malignant, benign, infectious, and inflammatory diseases among other applications such as cosmetic and other health-related aspects. Obviously, the hazards of this indiscriminate use started to appear. Only in the late 1930s, the ionization effect of radiation started to be better understood and it was this effect that became the basis for all future radiological dose measurements, allied with the studies of the biological effects of these radiation doses, called radiobiology.

Therefore, from a simple surface application of radiation with the evaluation of the effects based on the redness of the skin to a better knowledge of the dose–effects relationship of this radiation, almost half a century passed.

The first treatments were based on anatomical superficial surrogates and bone anatomy through X-ray images to define the target. Also, low-energy equipment (kilovoltage—kV) was used, which delivered a higher dose throughout the beam pathway and the skin surface, causing varied grades of skin reactions. To bypass this effect, the actual prescription dose was split into different radiation fields that were also defined according to surface anatomy and X-ray images. As the target or tumor location was set only indirectly, large margins were needed to avoid geographic miss and treatment failure. Later, megavoltage (MV) machines were developed and allowed delivery of higher doses to the target while better sparing the skin. Nevertheless, the same strategies for target definition were used for a long time. This irradiation technique may be called "conventional" or bidimensional and is still useful and used in many centers, mainly in middle- and low-income countries.

Technological advances in imaging tools, software, and hardware have made it possible to assess anatomy and dose distribution in three, or even four dimensions, providing more precise treatments and consequently with a lower risk of normal tissue damage. It is the era of three-dimensional or volume-based radiotherapy.

## 3D-CRT, IMRT, VMAT, IGRT, and 4D-RT^1^

The concept of three-dimensional conformal radiotherapy (3D-CRT) technique emerged in the 1960s and was the greatest step forward in radiotherapy. Volumetric imaging and computing sciences developments made it possible to visualize and quantify volumes rather than planar images for the definition of treatment targets and organs at risk and allowed improvement in dose distribution within the target volume while better sparing the surrounding tissues.

New hardware and software technologies were incorporated and permitted to consider the heterogeneous density of different tissues (different radiation absorption rates in bones and air, for example), according to the number of Hounsfield units of computed tomography (CT). Graphics and three-dimensional reconstructions have allowed the creation of increasingly individualized treatments according to the pathology and anatomy of each patient. In the 1990s and 2000s, other imaging data were integrated into planning systems (magnetic resonance imaging (MRI) and proton emission tomography (PET)). It then became possible to modulate the radiation delivery to better spare organs at risk of unnecessary irradiation. Combined with the creation of complex mathematical algorithms for inverse planning, this led to another major advance in radiotherapy, which is intensity-modulated radiotherapy (IMRT), when each tumor voxel is considered an individual target. Initially, with a rather slow dose delivery, the treatment became faster with the implementation of volumetric modulated arc therapy (VMAT).

With increasing precision, dose delivery needed to be assured. Real-time imaging systems were developed, such as oblique orthogonal radiographs performed in the treatment room and a CT scan coupled to the linear accelerator that can perform images while the patient is positioned in the treatment couch (cone-beam CT) to solve this issue. This strategy was called image-guided radiation therapy (IGRT), and even more precise technologies became available.

Simultaneously, and with the same concern for keeping high precision and treatment quality, four-dimensional (4D) CT scans, which account for respiratory motion, have been valuable for treating tumors that move with respiratory motion (e.g., lung and liver tumors), thus introducing the 4D radiotherapy (4D-RT) technique. When combined with IGRT, the level of precision allows the reduction of safety margins and increase of dose per fraction, thereby reducing the total number of fractions and giving rise to the concept of stereotactic body radiation therapy (SBRT) or stereotactic ablative radiotherapy (SABR).

## SRS, and SABR/SBRT

Despite being an undeniable contribution to the technological evolution of using ionizing radiation for therapeutic purposes, radiosurgery (SRS) dates back to the 1950s when Lars Leksell, a neurosurgeon, developed a non-invasive method for destroying intra-cerebral lesions that were inaccessible through conventional neurological surgery^1^. It is worth noting that at that time, three-dimensional CT images were not available, so the treatment was limited to conditions that could be assessed through angiography, such as arteriovenous malformations, and the treatment location was defined by stereotaxis.

Radiosurgery, by definition, is the delivery of high doses of radiation to a specific volume in a single fraction. In general, multiple fields with lower doses converge at a single center, where a high dose is concentrated, and the dose rapidly decreases at the periphery of the lesion. As the size of the lesion to be treated increases, this dose falloff becomes smaller, potentially limiting the technique, mainly for lesions larger than 4–5 cm. In these cases, the treatment is administered in more than one fraction, when radiosurgery is then referred to as fractionated stereotactic radiotherapy.

SBRT or SABR is the technology that has presented the larger growth in recent years. It follows the same principles as cranial radiosurgery, namely, delivering a potent, ablative, or nearly ablative dose in a few fractions (five or fewer fractions). SBRT can be performed using any of the technologies already described (e.g., 3DCT, IMRT, VMAT, IGRT, and 4D-RT) with the goal of delivering a high dose while sparing the surrounding normal tissues. It has proven to be an interesting technique for treating early lung tumors and oligometastases, particularly because it is effective and allows for a shorter treatment period. This technology also allowed for curative intent radiation treatments that changed clinical practice. For early-stage lung cancer^2^ and hepatocellular carcinomas^3^, SBRT has proven to be a standard of care for patients with curative intent. It has also been used in patients with oligometastatic cancers, with promising results^4^.

**Figure 1:**
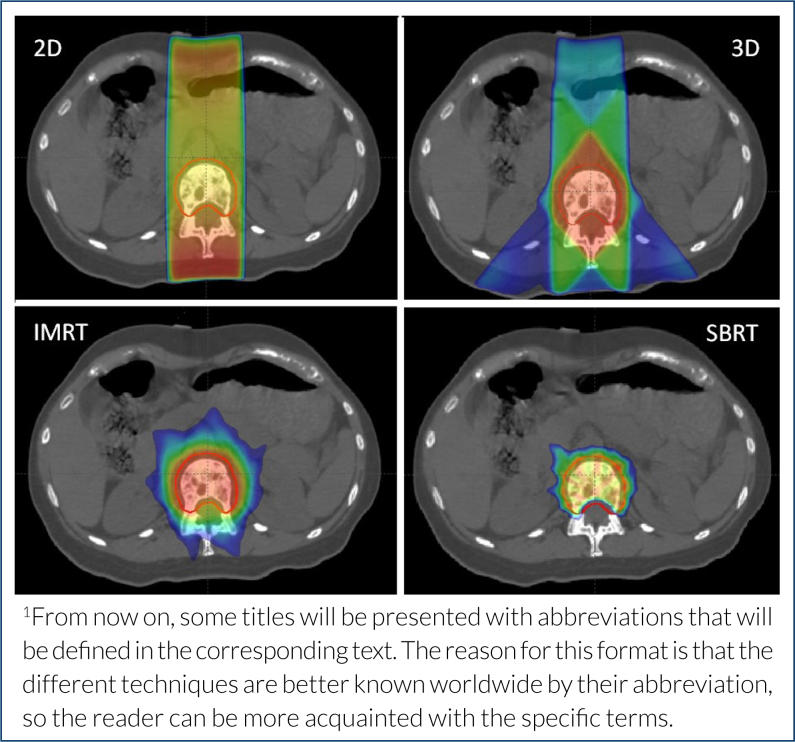
Comparison of radiotherapy techniques in the same case of spinal metastasis. Hot colors (red, yellow) illustrate higher doses (prescribed dose), and cold colors (green, blue) illustrate low-dose areas. The red line defines the target. Note that the more advanced the technique (from 2D to 3D, IMRT/VMAT, and SBRT), the higher the normal tissue sparing, including a very precise sparing of the spinal cord with SBRT, where very high doses are delivered (white arrow).

Figure 1 shows a comparison of the techniques’ evolution.

## ADAPTIVE RADIOTHERAPY

Tumor behavior during treatment, i.e., response or progression, can be mapped, and the radiation treatment can be adapted along the treatment, periodically: adaptive radiotherapy. Dose delivery can be changed and adapted according to the daily presentation or location of the lesion, to better assure the precision of dose delivery. However, not only the anatomical changes can be considered but also the biological behavior of the tumor during treatment. The concept of multidimensional radiotherapy (MD-CRT) based on the "Biological Target Volume" (BTV) was introduced in 2000 by Ling et al.^5^ and considers that the tumor has different active areas. These areas may be identified in functional exams (e.g., PET and scintigraphies), and thus, dose delivery can be shaped according to the areas that are more or less active at first, and if indicated, during treatment. This strategy has the potential to achieve better clinical outcomes. However, due to the complexity of the technique and availability of the method, it is not yet used worldwide and is the subject of many ongoing studies.

## 3D-IGABT

Brachytherapy is the radiation treatment where radioactive material is placed close, or in direct contact, or even inside the target lesion. This technique has also been described since the late 1800s and early 1900s, with several indications and applications. Gynecological (cervical and endometrial cancers) and prostate cancers are the ones where the technique is more often used nowadays. Remarks should be made mostly regarding the evolution of 2D brachytherapy, based only on indirect anatomic references available through simple X-rays, to 3D image-guided adaptative brachytherapy (3D-IGABT). The same principles used in volume-based external beam irradiation are now used for 3D-IGABT, where ultrasound, CT scans, and MRI images are used for treatment planning. The simple technical improvement led to more benefits than the association with chemotherapy, mainly for cervical cancer, where lower toxicity (acute and late) and survival improvement may be expected^6^.

## IORT

Intraoperative radiation therapy (IORT) is a treatment modality where radiation is delivered during surgery with the displacement of normal tissues away from the irradiated area. It can be performed using external beam radiation therapy equipment or brachytherapy. The main indications for IORT are for abdominal tumors surrounded by intestinal loops, and early-stage breast cancer, either as a single approach or combined with external beam irradiation^7^.

## HEAVY PARTICLES

Heavy particles can be neutral (neutrons) or charged (electrons, protons). Electrons are lightweight, negatively charged particles produced in the same treatment units as photons (linear accelerators). However, heavier particles require a very expensive infrastructure to be generated.

Among heavy particles, protons have the most prominent role in clinical practice. Due to the physical characteristics of these particles, the beams are particularly useful in treating structures deeply located within tissues while sparing structures surrounding the target volume that would interact with the radiation before reaching this volume.

Clinically, this allowed for the treatment of diseases that previously were treated with more modest outcomes, like chordomas of the base of the skull^8,9^, or far more toxic results, as in lymphoma patients^10^. Particularly for pediatric patients^11^, the use of proton therapy has proven to be highly effective and changed clinical practice where it is available.

This technology has been growing. However, despite numerous studies being published, the high cost of equipment and its large dimensions are significant drawbacks to the widespread use.

## FLASH-RT

It is worth mentioning the FLASH technique, which is defined as radiation therapy delivered at an ultrahigh dose rate (≥40 Gy/s), resulting in treatment times 400 times shorter than conventional treatments. It is promising in terms of its high anti-tumor effect and better preservation of normal tissues. FLASH-RT was first used in humans in Switzerland in 2018^12^ but remains an experimental treatment. However, it could become one of the primary radiation therapy technologies in clinical applications in the future.

## COMBINED TREATMENTS

Different combinations of irradiation with systemic treatments (e.g., chemotherapy and hormone therapy) have been proven beneficial for patients. New targeted and immunotherapies are emerging, with promising results; some of them are combined with radiation treatment^13^. Identification of genetic mutations and molecular tumor profiles is increasing and will provide a better patient and treatment selection, for individualized approach.

Therefore, it is expected that the combination of all these advances will give better results and more hope for the population.

## CLINICAL ASPECTS

All the technological advances in radiotherapy allowed, at first, better normal tissue sparing while providing better tumor coverage by the prescribed dose. Furthermore, target dose increments with higher precision and safety were possible, and better clinical results, with impact on local control and survival and less toxicity are being progressively achieved.

Allied with that, changes in radiobiological paradigms such as the sensibility of some tumors to radiation, such as breast^14,15^ and prostate cancers^16^, determined the development of successful hypofractionated radiotherapy regimens (higher dose/fraction with lower number of fractions)^17^, with many advantages among better management of overloaded departments and more convenience for the patients^18^.

In summary, the impacts of the radiotherapy advances in clinical practice involve the following:

- Improved treatment outcomes: Enhanced precision and accuracy in radiation delivery have led to improved tumor control rates and reduced toxicity, ultimately enhancing patient outcomes.
- Personalized medicine: The ability to tailor treatment plans based on individual patient characteristics and tumor dynamics has resulted in more personalized and effective treatments.
- Reduced side effects: By sparing healthy tissues and organs, modern radiotherapy technologies have significantly reduced the incidence and severity of treatment-related side effects, improving patients’ quality of life.
- Expanded indications: Many of these innovations have expanded the range of treatable tumors, allowing for more comprehensive cancer care.
- Shorter treatment times: Techniques such as SBRT and FLASH radiation therapy offer shorter treatment durations, reducing the burden on patients and healthcare systems.

## FINAL REMARKS

Technological advances in radiotherapy have revolutionized the field, offering clinicians a wide array of tools to treat cancer with unprecedented precision and efficacy. From IGRT to novel approaches like FLASH radiation therapy, these innovations continue to transform clinical practice by improving treatment outcomes, personalizing care, and minimizing side effects. As the landscape of radiotherapy technology continues to evolve, patients can expect even more refined and effective cancer treatments in the future. Radiotherapy remains a cornerstone of cancer care, and its future is brighter than ever.
